# Centripetal and Centrifugal Motions in Animals

**Published:** 1881-09

**Authors:** 


### CENTRIPETAL AND CENTRIFUGAL MOTIONS IN ANIMALS.

In a memoir published in the *Revue Scientifique*, last June, on "Writing Regarded from a Physiological Point of View," the author, M. Carl Vogt, after a lengthy discussión of centripetal writing (from right to left) and centrifugal (from left to right), drew the conclusion that the direction of the lines does not depend upon a physiological necessity, but only upon external conditions. Dr. G. Delaunay, who has for a long time been making researches on the same subject, has an article in the recent number of the same journal in which he endeavors to prove, on the contrary, that writing, as well as all motions and gestures in general, are dependent upon a physiological, and consequently an anatomical necessity.

The motions of quadrupeds can only take place horizontally or laterally ; yet there are a few that perform centripetal movements—the cat, for example, which strikes with its paw by bringing the latter toward the axis of the body. Monkeys make centripetal motions mostly ; but these animals hold a place between quadrupeds and man. Man alone is capable of making centrifugal motions. The physiolodical evolution of motion, which are successively vertical, then lateral and centripetal and then centrifugal in measure as we proceed from quadrupeds to the human species, is only the result of an anatomical evolution. According to Dr. Delaunay's researches, motions are rather centripetal than centrifugal in primitive or inferior races. A centripetal motion in a primitive race becomes centrifugal in measure as that race evolutes. Sanskrit, Persian and Greek were written from right to left before being written in the opposite direction. So our chronometers were wound up from right to left, before they began to be wound in the other direction. The English, however, are behind the age in this respect, since in the screws manufactured by them the threads still run from right to left, and most of their watches, like those of our ancestors, are wound from right to left. On the other hand, the people of the United States, who are in great part transformed English, and who without doubt are more advanced in evolution than those in Europe, use watches only which are wound from left to right and repudiate the old system still in use in England. Writing was centripetal among the ancient inferior races and is still so among those of modern times : Semitic, Phenician, Hebrew, Assyrian, Arabic, Chinese, Japanese, Negro, etc. Among the superior races not only is writing executed from left to right, but plans, sketches, shading etc., are begun in the same manner. A circle is always drawn centrifugally, that is, in the direction of the hands of a watch. In our designs and on our monuments the symmetrical ornaments are, starting from the median line, centrifugal. To consider other motions: we turn a door knob, door key, screw, stopcock, corkscrew, as well as tools for drilling, cranks of mills, wheels, etc., from left to right. In all trades and professions work is performed in a certain direction, which is generally centrifugal. To sum up, centrifugal motions, characterizing the superior races, are a sign of superiority marking the last term of evolution. As for sex, centripetal motions characterize a woman, while centrifugal motions are characteristic of man. A woman, for example, strikes with her palm, while a man gives a blow with the back of the hand. Every article of woman's clothing from the chemise to the cloak buttons from right to left, while man's garments button from left to right. When a woman puts on a man's coat she buttons it with the left hand, centripetally, doubtless being unable to button with her right centrifugally.

As for age, the motions of children are centripetal rather than centrifugal, therein resembling women.

From a psychological point of view centripetal gestures mark primitive, egoistic, retrograde ideas. On the contrary, centrifugal gestures express ideas and passions which are generous, altruistic, and expansive. From a psychological as well as from other points of views then, centripetal gestures characterize inferiority, and centrifugual superiority. As a result of his studies the author draws the conclusion that the centrifugal motions of abduction and of supination prevail in organisms most advanced in evolution, as the superior human races, men adults, intelligent beings, etc.; while, on the contrary, the centripetal motions of abduction and pronation predominate in individuals less advanced in evolution, as the inferior human races, women, children, people of little intelligence, monkeys, quadrupeds, etc.

Finally, the physiological evolution of motions, which is a consequence of the anatomical evolution of the limbs, proceeds from the centripetal to the centrifugal. Comparative anatomy and physiology then, explain why not only writing, but also other motions, are at first centripetal during the first phases of organic development, while the adductor muscles predominate over the abductor, and became centrifugal by very reason of the progress of evolution which bring about the predominance of the abductors over the adductors.

JABORANDI IN ASTHMA.—Dr. H. J. Thomas (*Chicago Med. Jour. and Exam.*) reports his experience in some twenty cases, in all of which he obtained good results. The fluid extract was used in four drop doses in the forenoon, afternoon and evening, and an eight or ten drop dose at bed time. In case the secretion of saliva was excessive the dose was lessened.

